# Back-Table Repair of Parenchymal and Collecting System Injury of a Living Donor Kidney

**DOI:** 10.7759/cureus.113899

**Published:** 2026-08-03

**Authors:** Joshua M Samaniego, Melissa L Barbosa, Marc Altimari, Mary Coomes, Oyedolamu Olaitan

**Affiliations:** 1 Surgery, Rush University Medical Center, Chicago, USA

**Keywords:** back-table repair, collecting system injury, donor nephrectomy injury, kidney graft salvage, living donor kidney transplantation, organ preservation, renal allograft injury, renorrhaphy

## Abstract

Living donor kidney transplantation provides superior outcomes compared with deceased donor transplantation, but unexpected allograft injury during donor nephrectomy can threaten graft viability. Reports describing back-table repair of iatrogenic donor kidney injuries are limited, particularly for full-thickness parenchymal defects involving the collecting system. We report a case of a 69-year-old male with end-stage renal disease secondary to type 2 diabetes mellitus and hypertension who underwent planned living-related kidney transplantation from his 43-year-old son. The donor underwent robotic-assisted left donor nephrectomy without an identified intraoperative complication. During back-table preparation, the allograft was found to have a large subcapsular hematoma and an approximately 2 cm parenchymal defect communicating with the calyces and renal pelvis. After clot evacuation and exposure of the injury, a three-layer repair was performed. The collecting system was closed with interrupted 6-0 polydioxanone sutures (PDS), the renal cortex and medulla were reapproximated with running 6-0 PDS, and the capsule was closed with running 6-0 Prolene with Surgicel as a pledget. The kidney was then implanted in the recipient’s right iliac fossa using standard vascular anastomoses and ureteroneocystostomy over a double-J stent. A perinephric drain was placed. The recipient had immediate graft function without the need for renal replacement therapy. Drain output was minimal, and the drain was removed on postoperative day 15. This case demonstrates that a structured multilayer back-table repair can salvage a living donor kidney with full-thickness parenchymal and collecting system injury. Careful injury assessment, multilayer reapproximation, and capsular reinforcement may support successful transplantation.

## Introduction

Living donor kidney transplantation (LDKT) offers improved graft survival and decreases time to transplantation compared with deceased donor transplantation [1]. LDKT requires meticulous donor nephrectomy and allograft handling to ensure optimal recipient, as well as donor outcomes [2]. Donor nephrectomy is generally safe in experienced centers, but intraoperative complications can occur. Iatrogenic allograft injury, particularly injuries involving the renal cortex or collecting system, poses an immediate threat to graft viability [3]. In a contemporary series of 425 open living donor nephrectomies, accidental donor-kidney injury occurred in five procedures (1.2%) [4], underscoring that this complication is uncommon but clinically relevant. Transplant teams must rapidly determine whether the kidney can be salvaged when such injuries occur. When such an injury occurs, the technique can be imperative to preserving the integrity of the kidney. Reports describing the management of iatrogenic puncture or laceration injuries to living donor kidneys are limited. Therefore, no standardized approach to repairing renal cortical injury currently exists.

Preoperative imaging demonstrated symmetric donor renal volumes and a single left renal artery, renal vein, and ureter. The right kidney had an accessory lower-pole artery; the left kidney was selected for donation based on its less complex vascular anatomy. The recipient had bilateral renal atrophy with suitable iliac vessels for transplantation. Transplantation-related injury may involve the renal parenchyma, collecting system, ureter, or vascular pedicle and can lead to hemorrhage, urinary leakage, obstruction, vascular thrombosis, graft ischemia, delayed graft function, infection, reoperation, or graft loss.

This report presents a case of LDKT in which the donor allograft sustained an iatrogenic puncture injury during donor nephrectomy. Prior literature is limited, as a few reports have demonstrated back-table repairs of kidneys from iatrogenic injuries [5,6], but not for a Grade IV donor kidney injury involving a full-thickness renal parenchymal defect, as presented in this case. A substantial parenchymal defect with extension into the collecting system was identified during back-table preparation. The kidney was salvaged using a layered reconstructive technique before implantation, which was followed by successful transplantation and uncomplicated graft function postoperatively. This case highlights practical considerations for a repair strategy that may be useful for similar events during renal transplant surgery.

## Case presentation

Patient background and clinical course

**Figure 1:**
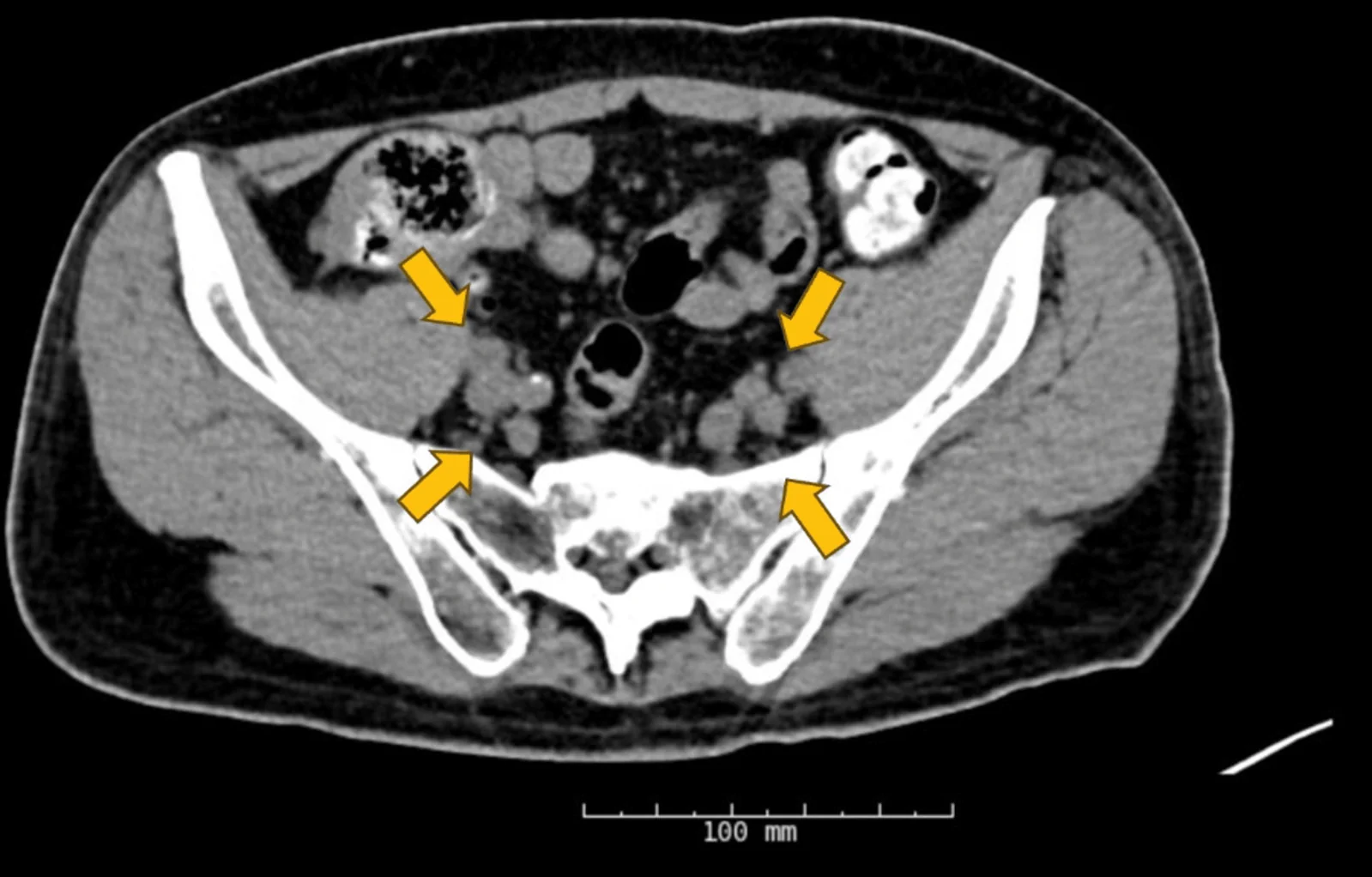
Preoperative abdominopelvic computed tomography of the recipient demonstrating suitable transplant vascular anatomy. Axial CT images of the recipient demonstrate the external iliac vascular regions evaluated preoperatively for transplant suitability, indicated by yellow arrows. No anatomic vascular contraindications to transplantation were identified.

A 69-year-old male with end-stage renal disease (ESRD) secondary to long-standing type 2 diabetes mellitus and hypertension presented for planned living-related kidney transplantation (LRKT). He had progressive chronic kidney disease with an estimated glomerular filtration rate (eGFR) <15 mL/min/1.73 m² but had not yet initiated dialysis. Additional comorbidities included coronary artery disease with prior stress-induced perfusion defects, managed non-operatively without revascularization. The patient has a pertinent family history of diabetes mellitus in both parents and stroke in the father. His medications included chlorthalidone, insulin aspart, amlodipine, losartan, atorvastatin, empagliflozin, and aspirin. Pre-transplant laboratory evaluation demonstrated elevated blood urea nitrogen, chronic normocytic anemia, intermittent hyperkalemia, and an elevated intact parathyroid hormone, consistent with secondary hyperparathyroidism (Table 1). Preoperative abdominopelvic computed tomography (CT) imaging demonstrated mild bilateral renal atrophy consistent with chronic medical renal disease, with no suspicious renal lesions, and suitable iliac vessels for transplantation without anatomic contraindications (Figure 1).

**Table 1:** Preoperative laboratory values of the kidney transplant recipient. Preoperative laboratory evaluation demonstrated severe renal dysfunction, consistent with end-stage renal disease and secondary hyperparathyroidism.

Laboratory Test	Patient Values	Typical Reference Range
Estimated Glomerular Filtration Rate (eGFR)	<15 mL/min/1.73 m²	≥60 mL/min/1.73 m²
Blood urea nitrogen (BUN)	105 mg/dL	7–20 mg/dL
Hemoglobin (Hgb)	8.3 g/dL	13.5–17.5 g/dL
Potassium	5.7 mmol/L	3.5–5.0 mmol/L
Intact Parathyroid Hormone (iPTH)	358.9 pg/mL	10–65 pg/mL

**Figure 2:**
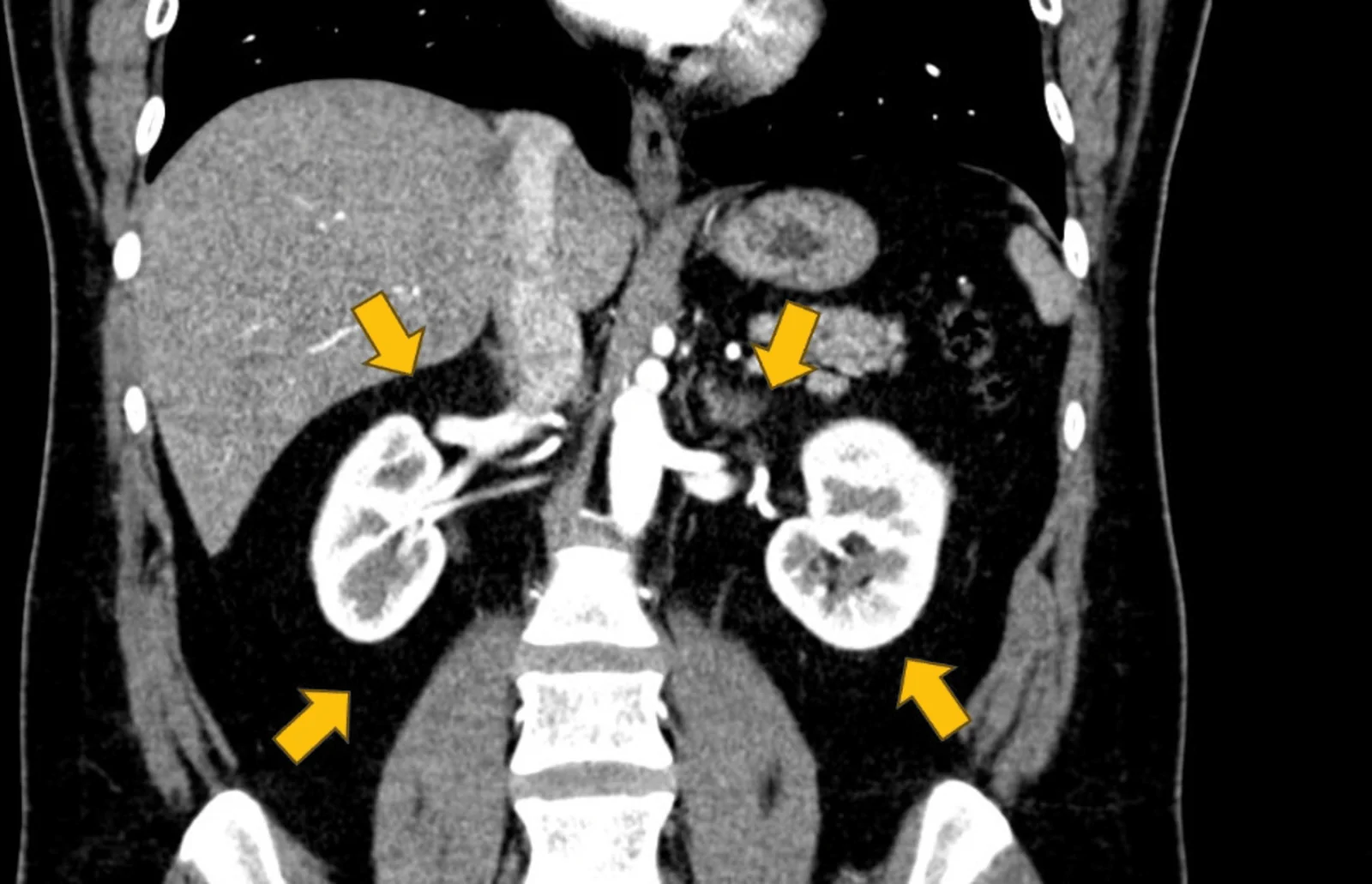
Donor computed tomography angiography demonstrating favorable renal vascular anatomy for living kidney donation. Coronal CT angiography of the abdomen and pelvis demonstrates symmetric bilateral renal enhancement and renal volumes, with the right kidney measuring 209 cc and the left kidney measuring 210 cc. The yellow arrows highlight the bilateral kidneys and renal hilar regions used to assess donor renal anatomy. Preoperative imaging demonstrated a single left renal artery, two right renal arteries including an accessory lower pole artery, and single renal veins bilaterally, supporting favorable vascular anatomy for donor nephrectomy planning.

The living donor was the patient’s 43-year-old son. He had no history of hypertension, diabetes, kidney disease, or cardiovascular disease. He was a former smoker with a 20-year history of smoking one to two cigars a day, which he quit in 2021. His body mass index (BMI) was 26.56 kg/m², and he had no prior abdominal surgical history. Baseline laboratory studies showed normal renal function, normal electrolytes, and a negative urinalysis without proteinuria or hematuria. CT angiography of the abdomen and pelvis demonstrated favorable renal vascular anatomy with a single left renal artery and two right renal arteries, including an accessory lower pole artery on the right, with single renal veins bilaterally and symmetric renal volumes, with the right kidney measuring 209 cc and the left kidney measuring 210 cc (Figure 2). Both kidneys demonstrated normal enhancement and excretion without hydronephrosis or hydroureter. The left kidney was selected for donor nephrectomy, given its less complex vascular anatomy. Final crossmatch testing was negative, and after multidisciplinary review, the donor was deemed an appropriate candidate for a robot-assisted left nephrectomy.

Operative course and injury repair

The donor underwent a robot-assisted left nephrectomy. Under general anesthesia, access was obtained through a 10-cm Pfannenstiel [GelPort (Applied Medical Resources Corporation, Rancho Santa Margarita, USA)] incision and three 8-mm robotic ports in the left upper and lower abdominal quadrants. The renal artery, renal vein, and ureter were subsequently identified and dissected. Intraoperatively, the kidney was mobilized while the vascular structures were dissected and divided without difficulty. The allograft was removed and flushed with approximately 700 mL of ice-cold preservation solution. The kidney flushed well and demonstrated uniform perfusion without areas of non-blanching. Estimated blood loss was minimal, and urine output was appropriate throughout the operation. No intraoperative complications or obvious injuries to the kidney were identified at the time of the donor nephrectomy.

**Figure 3:**
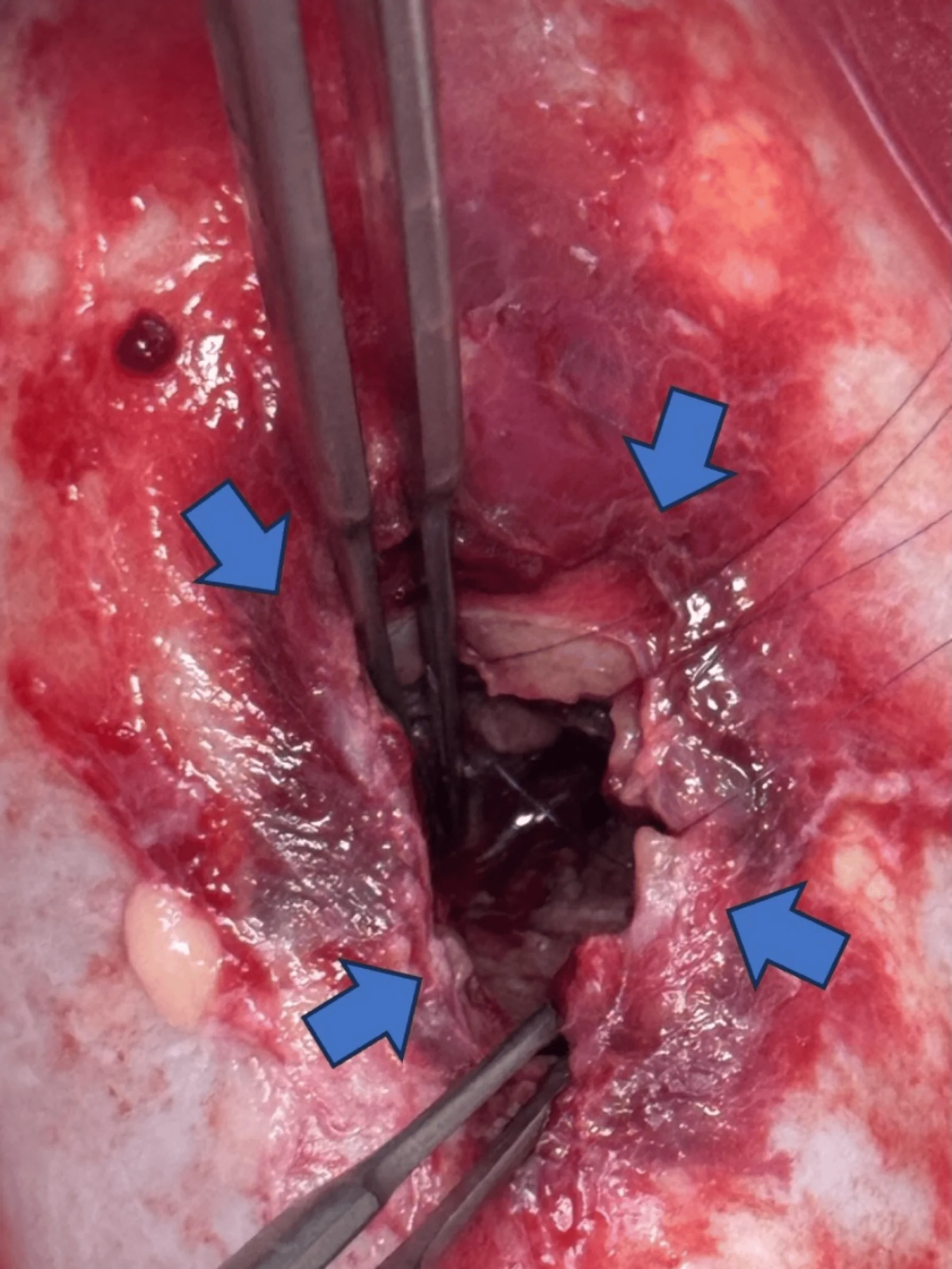
Intraoperative view of the donor kidney injury during back-table preparation. An intraoperative photograph obtained during back-table preparation of the donor kidney demonstrates a parenchymal defect with exposed collecting system communication. The blue arrows highlight the margins of the cortical/parenchymal injury and the surrounding renal capsule, delineating the area requiring back-table repair before transplantation.

**Figure 4:**
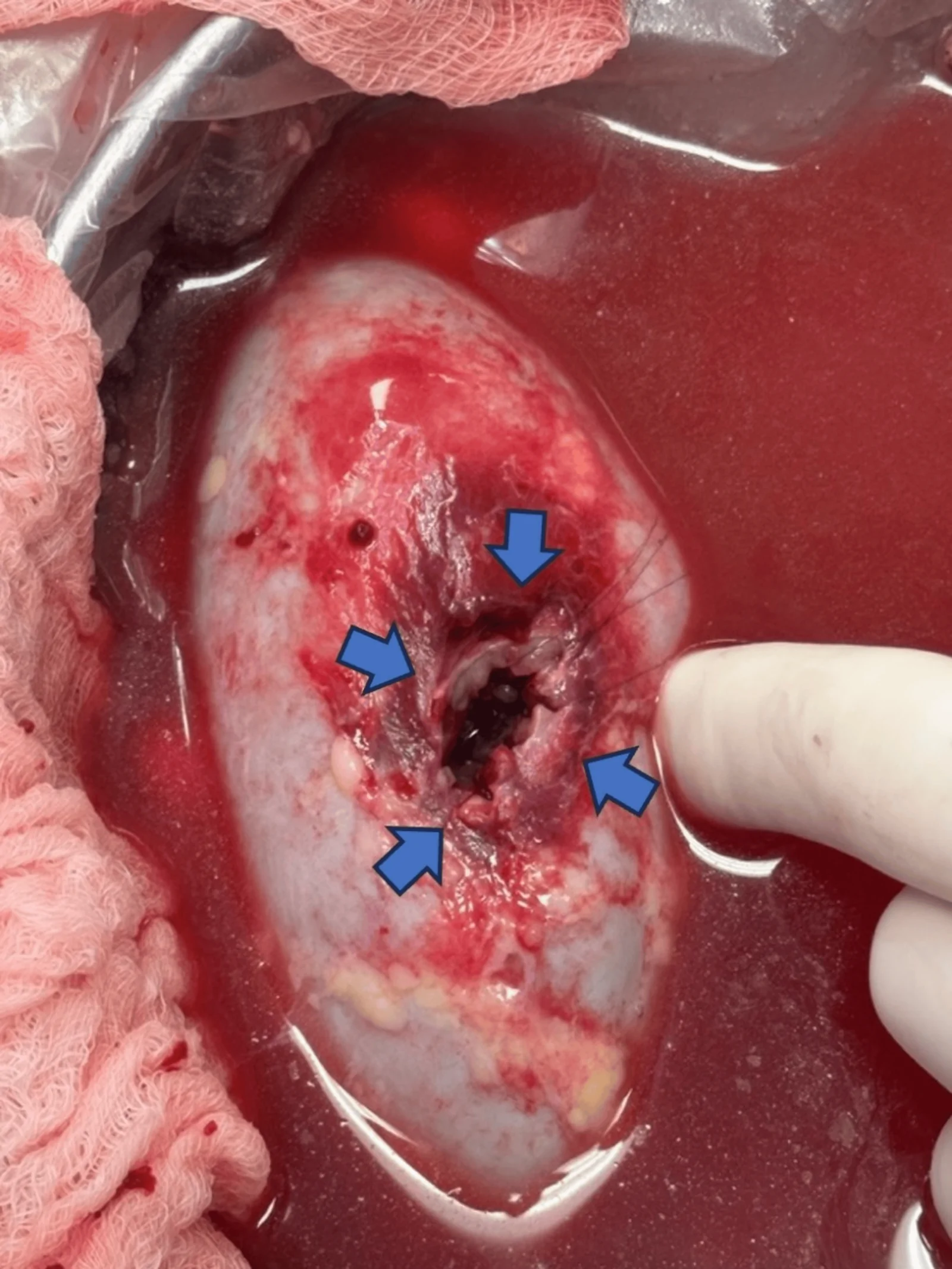
Another intraoperative view of the donor kidney injury during back-table preparation. Intraoperative photograph during back-table preparation demonstrates a cortical/parenchymal defect of the donor kidney with visible disruption extending into the collecting system. The blue arrows outline the margins of the renal parenchymal injury and identify the central defect requiring repair before implantation.

**Figure 5:**
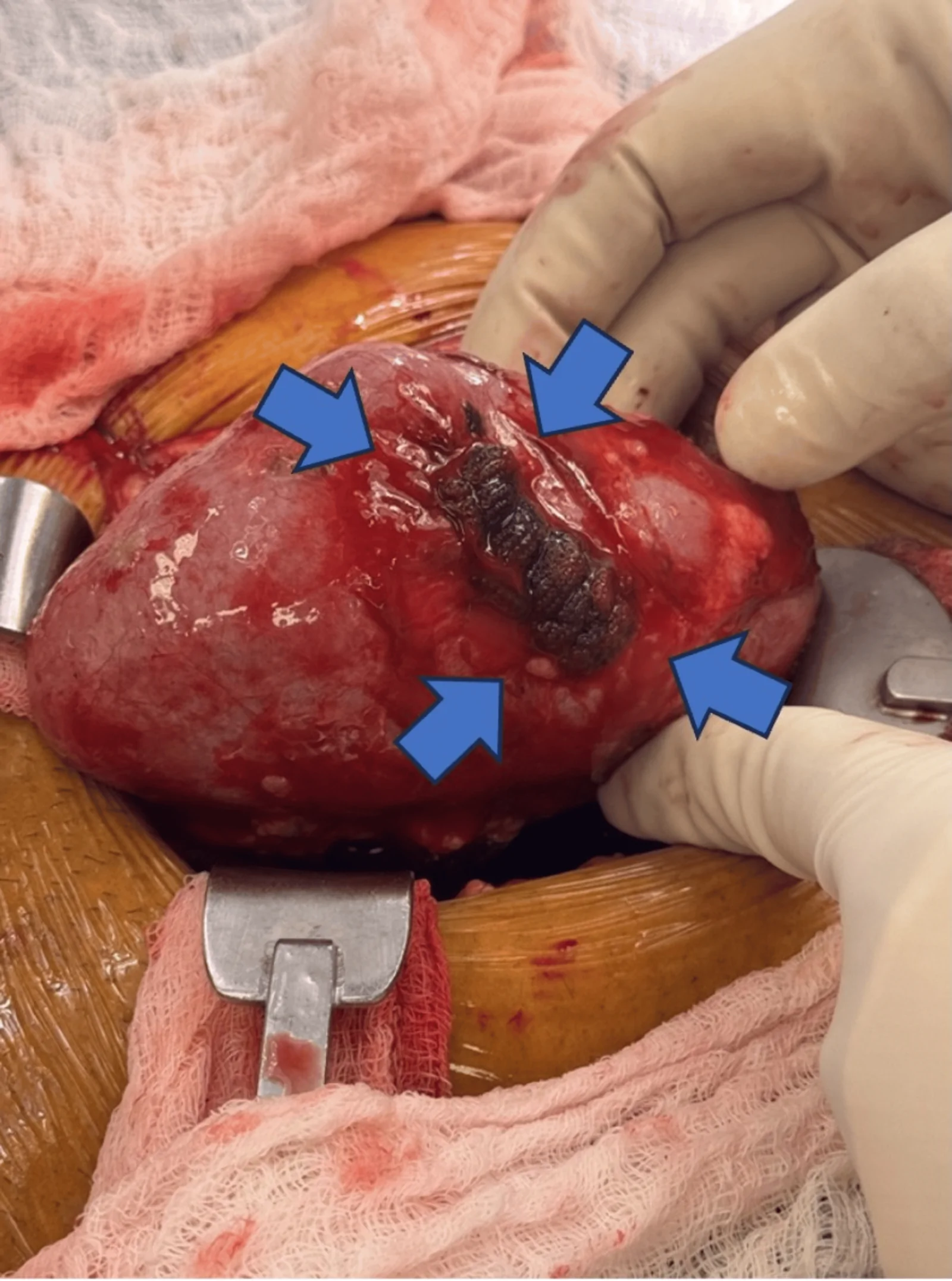
Intraoperative view of the donor kidney after multilayer back-table repair and organ reperfusion, demonstrating reapproximation of the renal parenchyma and capsular closure with hemostatic reinforcement. Intraoperative photograph after reperfusion demonstrates successful multilayer back-table repair of the donor kidney injury. The blue arrows highlight the repaired cortical/parenchymal defect, showing reapproximation of the renal parenchyma, capsular closure, and overlying hemostatic reinforcement at the prior injury site.

When the donor kidney became available, it was brought into the recipient room and placed on ice in cold preservation solution. During back-table preparation of the donor kidney, a large subcapsular hematoma was identified involving the renal cortex. Inspection revealed a cortical defect measuring approximately 2 cm with communication with the collecting system and renal pelvis (Figures 3, 4). The timing and mechanism of the injury to the donor kidney could not be determined. However, inadvertent puncture by an instrument outside the operative field of view during the robotic donor nephrectomy was considered a possible cause. The allograft required a complex repair. First, the adherent clot was evacuated to fully delineate the depth and margins of the defect. A portion of the overlying capsule was excised to optimize visualization of the cortical opening and its communication with the collecting system. Next, the involved calyces and collecting system were repaired using interrupted 6-0 polydioxanone sutures (PDS), carefully approximating the urothelial edges to restore watertight integrity. Afterwards, the renal cortex and medulla were re-approximated in a running fashion using 6-0 PDS sutures, restoring structural continuity of the parenchyma and obliterating dead space. The renal capsule was closed with 6-0 Prolene in a running fashion and reinforced with Surgicel for hemostasis. This completed a three-layer repair, consisting of two inner PDS layers and an external capsular Prolene layer (Figure 5).

The recipient underwent LDKT under general anesthesia, with thymoglobulin and methylprednisolone induction immunosuppression. The total operative time was 322 minutes, cold ischemia time was 117 minutes, and warm ischemia time was 28 minutes. The kidney was subsequently implanted in the recipient’s right iliac fossa via standard end-to-side renal vein to external iliac vein and renal artery to external iliac artery anastomoses. Initial reperfusion was slow but improved with time. Ureteroneocystostomy was performed using a Lich-Gregoir technique over a double-J ureteral stent. A surgical drain was placed adjacent to the graft because of the repaired allograft injury.

Post-operative course

The patient experienced immediate graft function without the need for renal replacement therapy. There was steady improvement in renal function, with serum creatinine declining from 7.14 mg/dL pre-operatively to 1.69 mg/dL at discharge on postoperative day four and further decreasing to a nadir of 0.70 mg/dL (Table 2). Tacrolimus was initiated and titrated to target serum levels of 7-9 ng/mL. During early outpatient follow-up, the patient developed a right gastrocnemius vein thrombosis, for which apixaban was initiated. Drain output decreased as expected, with fluid creatinine level similar to that of the serum, consistent with no urine leak. The surgical incision healed without complication. The drain was removed on postoperative day 15. Although drain output was minimal, the drain was kept in place until a few days after urethral catheter removal to ensure there was no leak. The patient had a urethral catheter that was removed on postoperative day seven and a double J stent that was removed at postoperative week six. The timing of the Foley catheter and double J stent removals was within our usual practice, but on the longer end compared to most patients. Overall, the patient remained clinically well with good graft function under close multidisciplinary transplant follow-up.

**Table 2:** Postoperative serum creatinine trend following living-related kidney transplantation. Serum creatinine decreased from 7.14 mg/dL preoperatively to 1.69 mg/dL at discharge on postoperative day 4, with further improvement to a nadir of 0.70 mg/dL, consistent with immediate and sustained graft function following living-related kidney transplantation.

Time Point	Serum Creatinine	Typical Reference Range
Preoperative	7.14 mg/dL	0.7–1.3 mg/dL
Discharge, Postoperative Day 4	1.69 mg/dL	
Postoperative Nadir	0.70 mg/dL	

## Discussion

The present case demonstrates that a structured back-table repair can effectively manage a sizeable injury to a living-donor kidney, thereby allowing successful graft utilization. Injuries involving the collecting system increase the technical complexity of transplantation and may raise concern for urinary leakage or other graft-related complications [7]. Nevertheless, meticulous repair permits safe transplantation without compromising graft function.

The principal contribution of this report lies in highlighting the translational application of renorrhaphy for donor graft salvage. While routinely used in renal trauma and nephron-sparing surgery, its use in injured transplant kidneys remains underreported [8]. The successful repair of both parenchymal and collecting system defects in this case demonstrates that the fundamental principles of renorrhaphy, including precise parenchymal closure and watertight reconstruction of the collecting system, are applicable in the transplant setting when vascular integrity is preserved [9].

Robotic-assisted living donor nephrectomy has expanded as a minimally invasive approach, but donor safety remains the central priority because the operation is performed on a healthy individual. As with other technically demanding procedures, robotic donor nephrectomy is associated with a learning curve related to hilar dissection, graft mobilization, instrument handling, retraction, and specimen extraction. During this period, even small technical errors or limited visualization can contribute to graft-related injuries, including capsular tears, parenchymal lacerations, or unrecognized puncture injuries. These risks highlight the importance of structured training and careful inspection of the allograft after extraction. In the present case, recognition of the injury during back-table preparation allowed for controlled assessment and repair before implantation, underscoring the need for transplant teams to be prepared to manage unexpected donor allograft injuries while maintaining recipient safety and graft viability.

In trauma literature, non-operative management (NOM) is appropriate for hemodynamically stable patients with renal injuries [10]. However, when operative intervention is necessary, renal preservation via renorrhaphy is the preferred strategy over nephrectomy [11,12]. McAninch et al. reported that approximately 89% of kidneys undergoing exploration were successfully salvaged using reconstructive techniques, with nephrectomy required in only 11% of cases [11]. Santucci and McAninch found that 69% of high-grade American Association for the Surgery of Trauma (AAST) grade IV renal injuries were repaired via renorrhaphy, with nephrectomy required in just 9% of cases [12]. Bjurlin et al. demonstrated that 38% of penetrating renal injuries were managed with renorrhaphy, achieving outcomes comparable to NOM, while nephrectomy was associated with higher morbidity [13]. Current guidelines from the American Urological Association (AUA) and the Eastern Association for the Surgery of Trauma (EAST)/American Association for the Surgery of Trauma (AAST) emphasize renal reconstruction whenever surgery is indicated, reserving nephrectomy for non-salvageable or exsanguinating kidneys [10,14]. This trauma literature serves as a framework for the feasibility of renorrhaphy to preserve renal function in the context of mechanical kidney injuries sustained during transplant operations.

Prior literature describing back-table repair of iatrogenic donor kidney injuries remains limited, likely because these events are uncommon, variably recognized and reported, and primarily documented as isolated successful case reports. Technical approaches also differ because injury patterns vary in depth, location, and involvement of the vasculature or collecting system, and no standardized repair guidelines currently exist. Rivera et al. described repair of a donor kidney parenchymal injury with decapsulation and renal pelvic exposure using several Monocryl sutures, perirenal fat patching, and ureteral stenting [5]. Meanwhile, Mohamed et al. reported successful graft salvage after parenchymal laceration during donor nephrectomy [6]. However, these reports do not describe a full-thickness cortical defect with direct collecting system involvement repaired using a defined multilayer reconstruction. In the present case, the repair was performed in sequential layers, with interrupted absorbable sutures used to close the collecting system, running absorbable sutures used to reapproximate the renal cortex and medulla, and a running nonabsorbable capsular closure reinforced with hemostatic material. This sequential three-layer approach, with independent repair of the collecting system, cortex and medulla, and renal capsule, may provide a reproducible framework for managing similar injuries. Thus, the principal contribution of this report is the combination of injury severity and a reproducible, layer-specific reconstructive strategy, which has not been described in other reports.

Salvaging a living donor kidney carries particular clinical and ethical importance, given organ scarcity and the benefits to recipients. Living donor transplantation offers superior graft survival and functional outcomes compared with deceased donor kidneys [15]. In 2023, 27,332 kidney transplants were performed in the United States, with 23% from living donors [16]. Five-year patient survival was consistently higher for living donor recipients compared with deceased donor recipients: 97.9% vs. 95.7% for adults aged 18-34 years and 84.4% vs. 72.5% for adults aged ≥65 years. Five-year graft survival similarly favored living donors: 90.0% vs. 82.2% for adults aged 18-34 years and 80.2% vs. 66.1% for adults aged ≥65 years [16,17]. These data underscore that optimizing living donor graft utilization enhances long-term outcomes and resource efficiency.

This case provides insight into intraoperative decision-making when donor graft injury is encountered. Assessment of injury severity and reparability enabled a reconstructive approach rather than discard of the graft. The back-table environment allows precise repair before implantation, optimizing hemostasis and reducing intra- and postoperative complications [18]. Secure collecting system closure minimizes urinary leakage, adequate cortical closure prevents post-reperfusion bleeding, and minimizing additional ischemia preserves graft function [19].

Although literature describing similar reconstructive approaches in kidney transplantation is limited, available evidence suggests that renal allografts requiring parenchymal repair can achieve satisfactory early function without increased complication rates [20]. This case reinforces that combined parenchymal and collecting system injuries do not preclude graft implantation and can be managed successfully with meticulous repair.

This report is intended to share our experience with a structured back-table repair approach for an unexpected full-thickness donor kidney injury involving the collecting system. This case highlights a feasible approach for salvaging an injured donor allograft and expanding the opportunity for successful transplantation when appropriate.

## Conclusions

Surgical repair of cortical and collecting system injuries in living donor kidneys can allow successful graft salvage and transplantation when performed with careful assessment of injury extent, meticulous layered reconstruction, and attention to technical principles. Although prior literature has described back-table repair of iatrogenic donor kidney injuries, reports involving full-thickness parenchymal defects with collecting system involvement remain limited. This case demonstrates that a structured back-table repair using collecting system closure, parenchymal reapproximation, and capsular reinforcement can preserve graft viability and support uncomplicated postoperative graft function. This approach may help maximize organ utilization after unexpected donor allograft injury.
